# Medical News and Miscellany

**Published:** 1889-02

**Authors:** 


### MEDICAL NEWS AND MISCELLANY.

WE REGRET exceedingly to chronicle the death of Dr. J. D. Beck's bright little daughter Bessie, who died at her father's home, in Mason, January 22d. She was a most promising child, a universal favorite, and her death is a terrible affliction to her parents. We tender them our heartfelt sympathy.

DR. J. S. DORSET was re-elected superintendent of the State Lunatic Asylum by a bare majority. Funny world; it was insisted by himself, the Governor and his other friends, that it required the full vote of the Board to put him out, and he refused to resign on the invitation of a majority. A poor rule that will not work both ways.

THE following Texas physicians are now at the New York Polyclinic: Drs. R. C. Brown, Waco; O. T. Halbert, Waco; A. V. Doak, Taylor; T. H. Wilson, Rockdale; A. C. Walker, Rockdale; N. T. Rope, Whitesboro; T. C. B. Justice, Ashville.

The largest class of post graduates ever assembled in any clinic is now in attendance upon the New York Polyclinic; the new hospital is said to be a big success. Wyeth is a great magnet (magnate) in surgery.

---

DR. R. P. TALLEY, of Belton, was badly hurt recently and is on crutches. Riding on horseback over one of the beautiful Bell county roads not navigable with buggy, his horse shied at an ox-team, and being ambitious, like his rider, he attempted to jump over the pole between the steers and the wagon. In doing so, Dr. Talley's leg was badly crushed against the wheel. We are pleased to see the doctor is able to be about, even though obliged to go on crutches.

---

DIED.—Doctor Zebulon McKenzie, a well known practitioner, of Galveston, was found dead in his bed by his landlady at an early hour on the morning of February 5th. An inquest was held and it was found that death had been caused by morphine taken accidentally. Doctor McKenzie was a native of St. Johns, New Brunswick, and about 49 years old. His practice was not lucrative, and he left nothing but a few old medical works, which will be raffled off by his friends for funds to defray his funeral expenses.—Telegram to the Statesman.

---

DR. WAY, whose card will be found amongst our new advertisements, comes to Austin most highly recommended by the leading specialists of New York, not only as a gentleman without reproach, but as a skillful and experienced Ophthalmologist. He served two years in Manhattan Eye and Ear Infirmary and six months as House surgeon, the latter just prior to the lamented Walton's service. He was office assistant to Dr. Agnew many months, filling the doctor's appointments in his absence. His practical training, together with a thorough course at the Post Graduate Medical College and the Polyclinic surely should entitle him to claim a knowledge of his profession, diseases of the eye and ear. Dr. D. B. St. John Roosa, Dr. David Webster, and other distinguished medical men of New York, endorse the doctor as worthy in every way of the confidence of the afflicted, and of the courtesies of the profession. We welcome him to Austin.

---

OZONE WATER IN CANCER.—Dr. F. Smidt, of Bavaria, says the parenchymatous injection of ozone water retards the growth of cancerous nodules, and causes their disappearance. He reports two cases: A man, age 60, epithelioma of lip. Had been removed twice in ten years. Returned again with marked cachexia. After four months treatment with ozone water the tumor had partly disappeared and partly been converted into a dense, hard mass. The ulcer had healed. The second patient, a man aged 56, suffered from an epithelioma at the inner angle of the eye of many years duration. Injections of ozone water were employed during two months, and effected a perfect cure, the ulcer being replaced by cicatricial tissue.

Smidt thinks the ozone destroys the cancerous masses without attacking the normal structures. He employs it in the strength of fifty milligrammes to the litre of water. Injections are made daily.

---

MARRIED.—On Thursday evening, January 17th, at the residence of the bride's mother, Mrs. Fribelie, by the Rev. Mr. Petot, Mrs. Mamie Lipscomb and Dr. J. W. Dupree, of Louisiana. Dr. Dupree is one of the most distinguished physicians of Louisiana, and carries off one of Tampa's most attractive ladies to his home in Baton Rouge, and with them go the best wishes of a host of friends for their happiness.—Tampa (Fla.) Tribune.

The JOURNAL extends its hearty congratulations to Dr. Dupree. He is one of our old time friends, and is worthy of all the blessings that good fortune can bestow. Dr. Dupree was one of the vice presidents of the late International Congress, and is a professor in the Louisiana State Agricultural and Mechanical College, an old Confederate Surgeon who passed his examination and was commissioned Surgeon (Major) at 21 years of age, and finally, a classmate of ours of the days of '60.

THE SUPREME COURT ON THE QUESTION OF THE REGULATION OF MEDICAL PRACTICE BY THE STATE.—An important decision has been rendered by the Supreme Court of the United States, supporting the right, claimed by the State Board of Health of West Virginia, to make rules for the regulation of the practice of medicine. The statutes of that state enact, that every practitioner must qualify himself for legal practice by obtaining from the Board a certificate that he is a graduate of a reputable medical cellege, or has been ten years in the state engaged in practice, or that he has passed a satisfactory examination before the Board. A practitioner named F. M. Dent, having been convicted of illegal practice, carried the case up to the State Court of Appeals, and thence to the United States Supreme Court, asserting that the law was unconstitutional, inasmuch as it deprived him of liberty and property without due process of law.—Medical News.

SUBSTITUTION BY DRUGGISTS OF MEDICINES PRESCRIBED.—If there is a practice among any repntable pharmacists or druggists that should be denounced, it is that of substituting the manufactures of some other druggist for that which is prescribed. It is none of the business of pharmacist or apothecary to dictate to the doctor what he should prescribe. If the honest apothecary has not the preparation the doctor has prescribed, and can not supply it, it is simply aud plainly his duty to say so, and not undertake to furnish a substitute without a free consultation with and the full consent of the doctor.

The apothecary concedes the whole thing whcn he says he supplies only Squibb's chloroform when this preparation is ordered. If he is honest in filling the prescription of the doctor strictly when that article is prescribed, why should he not be equally as honest when the preparations of Wm. R. Warner & Co., Rio Chemical Co., Battle, Peacock, Sharp & Dohme, Wyeth Bros., and many more reputable manufacturers.

Is it not dishonorable in principle to do otherwise.—Exchange.

THE INTELLIGENT COMPOSITOR and typographical errors have long been a standing subject for jest and ridicule, and many are the amusing instances of the pranks played by the types. In no profession or calling is greater care necessary than in that of type-setting. The appearance of a comma in the wrong place may reverse the meaning of a sentence, and the omission of a comma has so changed the meaning of a law as written, as to give rise to much annoyance and confusion, and even cause a suit at law. We could give illustrations of each of these instances. But what we intended to do, when this paragraph was begun, was to tell of an amusing prank of the types in getting out our last issue of the JOURNAL. The proof of an article was handed us, and we were horrified to read "Dr. Smith reported a case of cystitis patented October, 1884 !!" We sought the copy, and found the article read—"Dr. Smith reported a case of cystitis,—patient aet 74." "Shoot Polly as she flies, pop !" is quite familiar, as the work of a green hand in his first attempt to follow Geo. D. Prentice's copy; it read "Shoot folly as she flies.—Pope."

---

SURGEON GENERAL MARINE HOSPITAL SERVICE, AND THE JOURNAL A. M. A.—Congress has passed a bill which Dr. Hamilton says has been pending ten years, making the tenure of office of the supervising Surgeon General M. H. S. a life time appointment. Reynolds, of "Progress," says the bill increases the salary fifty per cent. Surely Dr. Hamilton is in good luck, and the Congressmen must think he is the right man in the right place there, if Reynolds does say he was not the right man in the right place as editor of the Journal of the American Medical Association. At any rate, Hamilton felt obliged to resign, and did resign as editor of the Journal, when he got news of his good luck in Congress. Who will succeed to the mantle of the great Davis? Meantime the Journal is conducted by the managing committee from the Board of Trustees of the Journal.

---

THE MISSISSIPPI VALLEY MEDICAL ASSOCIATION, at its last meeting, held in St. Louis, Mo., passed the following:

WHEREAS, The Board of Health of several of the states have inaugurated measures looking to the establishment of a higher standard of medical education, and

WHEREAS, It is of the utmost importance that some concerted action be taken by the profession looking to this end.

*Resolved*, (1.) That this Association appoint annually a standing committee of two from each state represented, to be known as the "Committee on Medical Education," whose duty it shall be to confer with all regularly organized Medical Societies and Boards of Health, with a view to enlisting their co-operation in promoting the passage of uniform laws in each state and territory of the Union, touching the period of study and literary acquirements of every candidate for graduation in medicine.

*Resolved*, That it be the duty of said committee to make an annual report in writing, settling forth the work done and the progress made.

Members for Texas: Drs. F. E. Daniel, (declined), and R. H. Chilton.

---

FLOWERS.—Everybody knows that we love flowers; the subject is a kind of hobby with us, and we hope to be excused for mentioning a subject thought to be irrelevant to medicine;—but really it is not, for does not the poet say, "each dying calyx is a *memento mori*, yet font of hope"? And if dying and death are not related, and closely related to medicine, what is? We trust the parallel extends also to the other part of the couplet where the idea of resurrection is suggested. And again the poet says,—"Each cup's a pulpit, and every leaf a book," and doctors are supposed to be fond of both, pulpits (?) and books! If they are not, they ought to be.

But to the point. We wish to render a service to all flower lovers, like ourselves, and in citing them to the best place to get the best plants and bulbs and seeds for the least money, we believe we do them a service. That place, in our judgement, is the great nursery of Charles A. Reeser, of Springfield, Ohio. We have received a large lot of the choicest Begonias, Camelias, Geraniums, etc., from him, and to say that we are abundantly satisfied, both with selection and price, would be but to feebly express our great delight; they arrived blooming! Send for his splendid catalogue. Make no mistake in his address.

SOMETHING ELSE NEW IN PHARMACEUTICALS.—Several of our manufacturing pharmacists are certainly valuable aids to the practicing physician, for so soon as a new idea or discovery is announced in therapeutics they set to work to facilitate its execution ; if a new drug appears they work it up in a form for administration, both convenient and efficient. They rob the administration of medicine of much of its disagreeable features. Park, Davis & Co. now announce a glycerine suppository for constipation, and in announcing it say ; "For a number of years glycerin has been successfully used in the form of enemata, to provoke alvine evacuation. Owing to the physical and chemical properties of this article, many difficulties have been encountered in the endeavor to present it in a suitable and convenient form which would dispense with injection apparatus. While it may be conveniently administered in rectal capsules its enclosure in gelatin is not permanent, as capsules containing it are rapidly attacked and become distorted, owing to the hygroscopic character of the glycerin and its solvent action on the gelatin, the greater part of the glycerin usually escaping. Neither does it appear possible to incorporate gelatin with the glycerin and produce a suppository having any activity, as such a composition necessarily requires a certain amount of water. This admixture of water interferes decidedly with the action of the glycerin. Whatever theories may be indulged in as to the rationale of its action, the best effects are obtained when only a minute quantity of water is present, in other words when the glycerin is nearly anhydrous."

"The effect would appear to be due to some direct chemical action of the glycerin on the rectal mucous membrane, possibly a combined stimulant and mechanical effect, the latter due to abstraction of water, or rather excitation of a watery discharge. Be that as it may, it produces in some way an activity reflex intestinal peristalsis. The remedy has been used in the most varied forms of intestinal and gastric troubles and is characterized by extreme promptness of action varying from a few minutes to half an hour, usually producing an evacuation when the suppository is only partially dissolved. '

"The conclusion arrived at, from an extensive employment, is, that their action while not infallible, is constant and free from danger. We now prepare suppositories containing 95 per cent. of glycerin in a form and size adapted for administration to both infants and adults."

Simultaneously with the above advance in therapeutics is announced by Sharp & Dohme, of Baltimore, nito-glycerine in soluble compressed tablets for hypodermic use. Nitro-glycerine is the remedy for many ills that kill quickly, such as angina pectoris, etc., and for emergencies like chloroform narcosis, cardiac failure from any cause, cases where prompt and effiicen action is absolutely necessary to save life. The trouble heretofore has been the difficulty of administration; to get the medicine to take effect, which it is slow to do when administered by the mouth, even when that is possible, and so far as we know it has never been possible, heretofore, to give it hypodermically. The intelligent physician will "catch on" at once, and prepare for such emergencies.

---

DIED.—In Brenham, Texas, February 10th (inst.), Dr. J. M. Ross, of that city. Dr. Ross was one of the oldest and most distinguished physicians of Central Texas, and his death is a sad loss to the profession and to the State. He was a member of the Texas State Medical Association and of the Physicians' Mutual Benefit Association, and for many years prominently identified with the Baptist Church.

---

POCKET MEDICAL FORMULARY, arranged therapeutically. By Alex. Hazard, M. D. Revised and enlarged by A. S. Gerhard, A. M., M. D., Philadelphia. With an appendix containing formulas and doses for hypodermic medication ; a table of eruptive fevers ; and poisons, their symptoms, antidotes, and treatment. Second edition. A. L. Hummel, M. D., Publisher, Philadelphia.

---

We are in receipt of the PHYSICIANS' POCKET REFERENCE BOOK and VISITING LIST for 1889, arranged and published by J. H. Chambers & Co., St. Louis, Mo. It is as well arranged as any visiting list on the market.

